# Genome Editing Targets for Improving Nutrient Use Efficiency and Nutrient Stress Adaptation

**DOI:** 10.3389/fgene.2022.900897

**Published:** 2022-06-14

**Authors:** Lekshmy Sathee, B. Jagadhesan, Pratheek H. Pandesha, Dipankar Barman, Sandeep Adavi B, Shivani Nagar, G. K. Krishna, Shailesh Tripathi, Shailendra K. Jha, Viswanathan Chinnusamy

**Affiliations:** ^1^ Division of Plant Physiology, ICAR-Indian Agricultural Research Institute, New Delhi, India; ^2^ Roy and Diana Vagelos Division of Biology and Biomedical Sciences, Washington University in St. Louis, St. Louis, MO, United States; ^3^ Department of Plant Physiology, College of Agriculture, KAU, Thrissur, India; ^4^ Division of Genetics, ICAR-Indian Agricultural Research Institute, New Delhi, India

**Keywords:** genome editing, CRISPR-Cas, nutrient stress, nutrient use efficiency, biofortification, abiotic stress

## Abstract

In recent years, the development of RNA-guided genome editing (CRISPR-Cas9 technology) has revolutionized plant genome editing. Under nutrient deficiency conditions, different transcription factors and regulatory gene networks work together to maintain nutrient homeostasis. Improvement in the use efficiency of nitrogen (N), phosphorus (P) and potassium (K) is essential to ensure sustainable yield with enhanced quality and tolerance to stresses. This review outlines potential targets suitable for genome editing for understanding and improving nutrient use (NtUE) efficiency and nutrient stress tolerance. The different genome editing strategies for employing crucial negative and positive regulators are also described. Negative regulators of nutrient signalling are the potential targets for genome editing, that may improve nutrient uptake and stress signalling under resource-poor conditions. The promoter engineering by CRISPR/dead (d) Cas9 (dCas9) cytosine and adenine base editing and prime editing is a successful strategy to generate precise changes. CRISPR/dCas9 system also offers the added advantage of exploiting transcriptional activators/repressors for overexpression of genes of interest in a targeted manner. CRISPR activation (CRISPRa) and CRISPR interference (CRISPRi) are variants of CRISPR in which a dCas9 dependent transcription activation or interference is achieved. dCas9-SunTag system can be employed to engineer targeted gene activation and DNA methylation in plants. The development of nutrient use efficient plants through CRISPR-Cas technology will enhance the pace of genetic improvement for nutrient stress tolerance of crops and improve the sustainability of agriculture.

## Introduction

Genetic improvement of crop abiotic stress tolerance and nutrient use efficiency (NtUE) has become indispensable due to the climate change vagaries and the constant challenge of feeding the burgeoning population. The availability of genetically robust resource use efficient genotypes could minimize the input cost and ensure sustainable food sufficiency (Zhu, 2002; Mahajan and Tuteja, 2005; Hirel et al., 2007; Balyan et al., 2016). Mutation led heritable variations are an important source for genetic improvement of crops (Olsen and Wendel, 2013) which have been exploited since the early phase of crop breeding (Tang et al., 2020). From the 1950s onward, induced mutations gained importance in varietal development. Due to their randomness, the conventional mutagenesis strategy is prone to several challenges including the screening of thousands of mutants to obtain desirable mutations (Miglani, 2017). The availability of genome editing technologies enabled precise and predictable genetic modifications in the plant genome to bring desirable changes of economic and environmental importance (Gaj et al., 2016).

Genome editing is a powerful tool applicable to every branch of life science for knock out, knock down, or alteration of gene expression without disturbing the genetic makeup of the organism (Ahmed et al., 2019). Genome editing can create a loss of function mutant of “susceptibility genes” or shut down a negative regulator of the stress response pathway (Feng et al., 2013), at the same time it can be employed to enhance the expression, activity and stability of positive regulators of the stress response pathway (Zhou et al., 2017). Theoretically, any gene in an organism can be manipulated with the help of sequence-specific DNA nucleases (Takasu et al., 2010). Mega nucleases, Zinc Finger Nucleases (ZFNs), Transcription Activator-like Effector Nucleases (TALENs), and Clustered Regularly Interspaced Short Palindromic Repeat associated Nuclease (Cas), are site-specific DNA nucleases developed over the past few years (Hisano et al., 2021).

Cas nuclease consists of a specificity governing DNA binding domain and a non-specific nuclease domain that creates a double-stranded break (DSB) on the target DNA, thus driving targeted modification of the genome (Zhang F. et al., 2020). The DSB in DNA is repaired by Non-Homologous End-Joining (NHEJ) or the Homologous Recombination (HR) mechanisms of the cell. Errors in NHEJ or changes in the repair template DNA in HDR (Homology Directed Repair) causes mutation (Chen et al., 2013). The NHEJ repair is an error-prone mechanism that fuses the broken DNA with minor additions and deletions of nucleotides (Kato-Inui et al., 2018). The HDR pathway uses a homologous donor DNA as a template to repair the DSBs precisely (Kato-Inui et al., 2018). Most plant genome editing experiments have taken advantage of NHEJ pathway for gene knockout and generating frameshift mutations. Before the discovery of CRISPR as a gene-editing tool, ZFNs were used for genome editing (Chandrasegaran and Carroll, 2016). A pair of ZFNs target a specific site, one recognizes the upstream sequence and the other one identify the downstream sequences of the target site to be altered (Miller et al., 2007; Szczepek et al., 2007). Similar to ZFNs, TALENs involve a fusion of a FokI nuclease domain with a DNA binding domain which is the TALE (Transcription Activator-like Effector) pattern adapted from the virulence factors of the plant bacterial pathogen *Xanthomonas* (Gaj et al., 2016). TALEN’s DNA binding domain has multiple repeating units with each unit spanning 33–35 amino acids that can recognize one DNA base pair (Joung and Sander, 2013). Though TALENs are efficient genome editors, it involves protein engineering depending on the target DNA sequence (Gupta and Musunuru, 2014). Recently a groundbreaking gene-targeting tool based on the RNA-guided Cas9 nuclease from the type II prokaryotic Cluster Regularly Interspaced Short Palindromic Repeats (CRISPR)-Cas was developed (Jinek et al., 2012b). The first instance of CRISPR/Cas was observed in 1987, as an adaptive immunity mechanism of bacteria to viral DNA (Ishino et al., 1987). In the natural system, CRISPR loci upon transcription produce CRISPR-RNA (crRNA) and trans-activating CRISPR-RNA (tracrRNA) which upon base pairing make functional guide RNA (gRNA). For genome editing, CRISPR loci are transcribed into a synthetic single-guide RNA (sgRNA). The gRNA/sgRNA forms a functional complex with CRISPR-associated nuclease (Cas9) and directs the nuclease to genomic loci based on the complementarity of a 20-bp stretch spacer sequence of sgRNA, cleaving it upstream of a necessary 5′-NGG Protospacer Adjacent Motif (PAM) (Xie and Yang, 2013). The *Streptococcus pyogenes* (*Sp*) Cas9 and Cas9 isolated from other organisms have different PAMs and have different distances to the active (cleaving) sites. CRISPR/Cas9 system utilizes gRNA that make a complex with Cas9 and make DSB at the target. Unlike ZFNs and TALENs which requires complex protein engineering for changing the DNA target sequence CRISPR/Cas requires a change of only 20 bp (Liu et al., 2017).

In the recent past, several strategies have been utilized to improve the nutrient response of crop plants, such as differential alternative splicing of genes in response to boron deficiency (Gu et al., 2019), overexpression of genes coding for enzymes of the ammonia assimilation (GS/GOGAT) pathway under constitutive promoters for improving nitrogen (N) use efficiency (Wei et al., 2018), exploiting the role of long non-coding RNA (lncRNA) in *nigt1.1 nigt1.2* double mutant for enhanced nitrate uptake under low P stress (Ueda et al., 2020), etc. But these approaches achieved no significant and stable improvement in NtUE. For example, transgenic plants overexpressing N assimilatory genes did not significantly enhance N Utilization Efficiency (Pathak et al., 2008; Lightfoot, 2009). This review outlines the potential of CRISPR-Cas technology in understanding and facilitating NtUE and nutrient stress tolerance.

### Genome Editing Targets for Improving Nitrogen Use Efficiency

**FIGURE 1:**
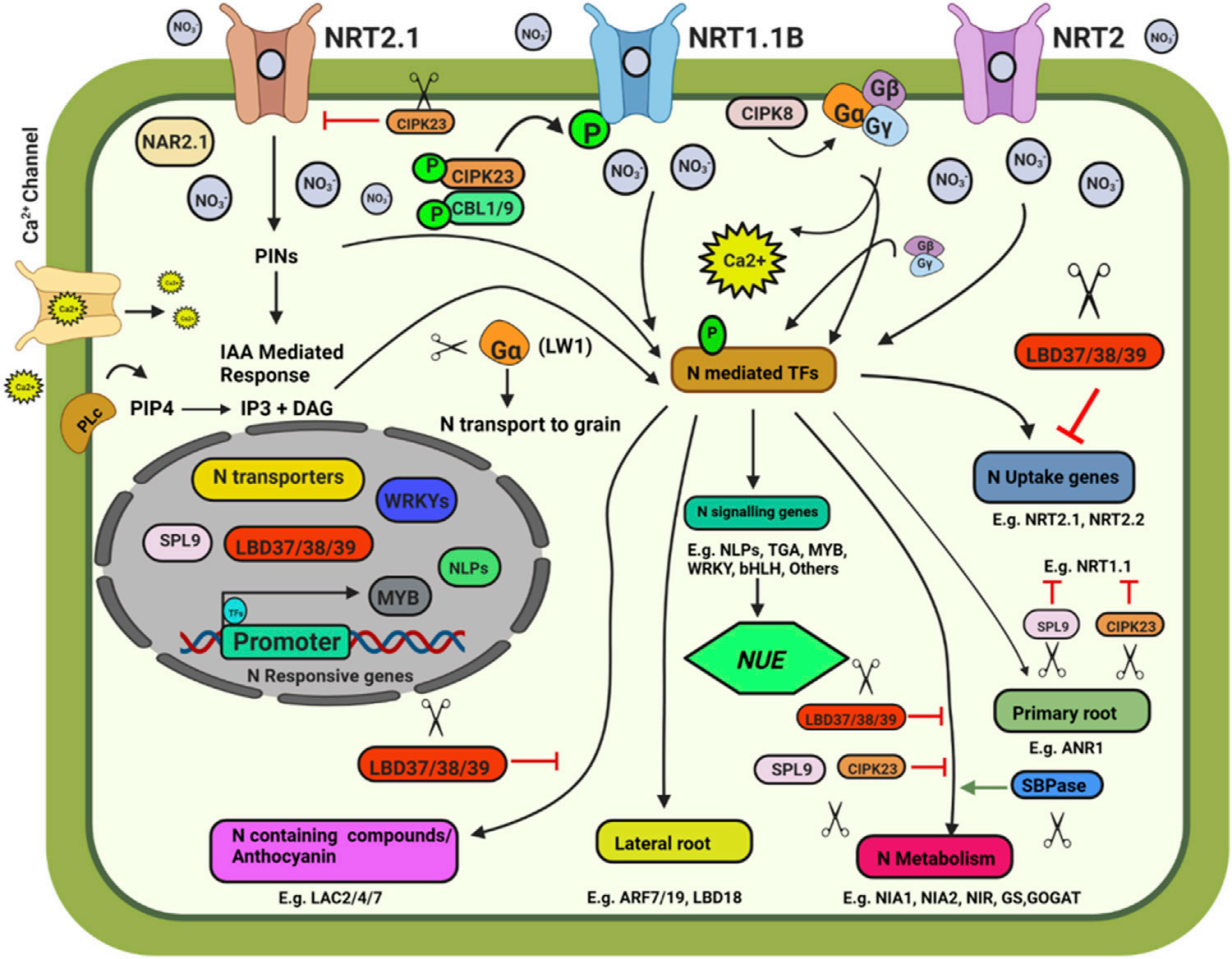
Schematic model depicting plant response to nitrate availability and signaling. Illustration was made with Biorender (https://biorender.com/). The negative regulators for targeted genome editing are indicated with a scissor sign.

Plants posess well-developed uptake and signalling systems to cope with soil N fluctuations. Nitrate induces several genes including its transporter families, NRT1 (Nitrate Transporter), NRT2 and its assimilation pathway genes, encoding Nitrate Reductase (NR), Nitrite Reductase (NiR) (Wang Y.-Y et al., 2018). Induction of these genes in NR-deficient mutants in response to nitrate indicates that nitrate *per se* is also a signal molecule (Zhao et al., 2018). Nitrate mediated activation of genes occurs very rapidly (within minutes), without the necessity for protein synthesis, is termed “Primary Nitrate Response” (PNR) (Krouk et al., 2010). PNR is regulated by various kinases, transcription factors, and noncoding RNAs. Phospho-proteome level changes are controlled by nitrate supply, and 773 unique phosphorylated peptides were identified within a short period of nitrate supply (Engelsberger and Schulze, 2012). As a signalling molecule, nitrate plays a crucial role in plant growth and development (Figure 1). Several developmental processes such as germination (Alboresi et al., 2005), root system architecture (RSA), leaf emergence, leaf growth (Guo et al., 2001; Rahayu et al., 2005), and flowering (Castro Marín et al., 2011) are responsive to nitrate availability.

The Site-Directed Nuclease 1 (SDN1) genome editing approach was found to be promising and can be used to knock out the negative regulators of N uptake and signalling pathways. Some of the potential targets are discussed below. Three members of the *LATERAL ORGAN BOUNDARY DOMAIN (LBD)* gene family of transcription factors (LBD37, LBD38, and LBD39) were identified as repressors of anthocyanin biosynthesis and N availability signals in *Arabidopsis* (Rubin et al., 2009). The overexpression of LBD37/38/39 could repress the expression of nitrate-responsive genes, including *NRT2.1*, *NRT2.2*, *NIA1*, and *NIA2*, indicating the negative role of the three LBD members in nitrate signalling. SQUAMOSA Promoter-Binding Protein-Like 9 (SPL9) and CBL-interacting protein kinase 23 (CIPK23) also negatively affect the nitrate response. SPL9 reduces the transcript levels of genes involved in N uptake, i.e., *NRT1.1*, *NIA2*, and *NIR*, while miR156 negatively regulates SPL9’s transcript level (Krouk et al., 2010). CIPK23 negatively regulates the expression of the high-affinity nitrate transporter *NRT2.1*. CIPK23 acts as a regulator for the high-affinity switch of NRT1.1 under low nitrate concentrations to enable NRT1.1 to operate as a high-affinity nitrate transporter by phosphorylating it at T101, thus playing an essential role in N uptake (Ho et al., 2009). MicroRNAs such as miR399 and miR827 play an indispensable role in maintaining N and Pi homeostasis in plants by targeting *NITROGEN LIMITATION ADAPTATION* (*NLA*) and *PHOSPHATE2* (*PHO2*) (Nguyen et al., 2015). Knocking out these miRNAs using CRISPR-Cas will help better understand their role in N and P homeostasis. Knocking out of Sedoheptulose-1,7-Bisphosphatase (SBPase), involved in carbon assimilation, could reduce the expression of NR, GS and GOGAT, and alter N metabolism in tomato plants (Ding et al., 2018). NIN like proteins (NLPs) are transcriptional regulators of nitrate signalling (Jagadhesan et al., 2020). CRISPR-Cas9 knockout mutant *OsNLP4* showed reduced NUE and yield (Wu et al., 2020). *OsNLP1* is rapidly inducted by N deficit. OsNLP1 genome-edited lines showed impaired grain yield and NUE under N limiting conditions (Alfatih et al., 2020)**.** The AtNLP7 promotes lateral root development by binding to *TRYPTOPHAN AMINOTRANSFERASE RELATED 2* (TAR2) promoter region in a nitrate dependent manner (Zhang et al., 2021b). In cotton, the overexpression of Arabidopsis *NLP7* enhanced the NUE and yield (Jan et al., 2022). CRISPR/Cas edited *OsNLP3* lines showed reduced plant growth, grain yield, and NUE under sufficient nitrate conditions, whereas in low nitrate or in ammonium supplementation, *osnlp3* mutants performed at par with the wild type (Zhang et al., 2022).

Map-based cloning followed by CRISPR-Cas9, gene-editing confirmed the involvement of Leaf Width 5 (LW5) in N uptake and utilization (Zhu et al., 2020). The *lw5* mutant showed higher rate of photosynthesis, higher chlorophyll content and higher N uptake rate, however the reduced grain nutrient remobilization results in small grains. The *abnormal cytokinin response1 repressor 1* (*HvARE1*) was identified as a regulator of NUE in a genome-wide association analysis. ARE1 was identified as a suppressor of plastidic Fd-GOGAT in rice. The *are1* mutant plants of rice, wheat, and barley showed improved yield and higher NUE, making it a worthy genome editing candidate (Wang et al., 2018b, 2021; Zhang et al., 2021a; Karunarathne et al., 2022). In wheat and barley, CRISPR/Cas9 gene-editing of *ARE1* enhanced NUE (Zhang et al., 2021a; Karunarathne et al., 2022). The GROWTH-REGULATING FACTOR 4 (OsGRF4) is a transcriptional regulator of numerous N metabolism genes that work in opposition to the DELLA growth repressor. CRISPR/Cas editing has shown that changing the OsGRF4-DELLA balance by increasing *OsGRF4* abundance improves NUE and grain production (Li et al., 2018a; Gao et al., 2020). Zhang J. et al. (2020) used a multiplex CRISPR/Cas9 vector to modify the *MIR396* gene family in rice, targeting MIR396a, MIR396b, MIR396c, MIR396e, and MIR396f at the same time. Two microRNAs, MIR396e and MIR396f, have been discovered to regulate grain size and plant architecture. The role of *miR396* genes in rice was investigated using CRISPR/Cas9 by knocking down MIR396e and MIR396f. MiR396ef mutants demonstrated a relative increase in grain yield with larger biomass under reduced N conditions and increased grain production under normal N conditions. The DELLA protein gene *SLR1* and nitrate transporter NRT1.1B were targeted using the CRISPR/Cas9 cytidine deaminase enzyme (APOBEC1) base editing system, resulting in point mutations and a C/T substitution (Thr327Met) in NRT1.1B, which boosted NUE in rice (Lu and Zhu, 2017).

### Genome Editing Targets for Improving Phosphorus Use Efficiency

**FIGURE 2:**
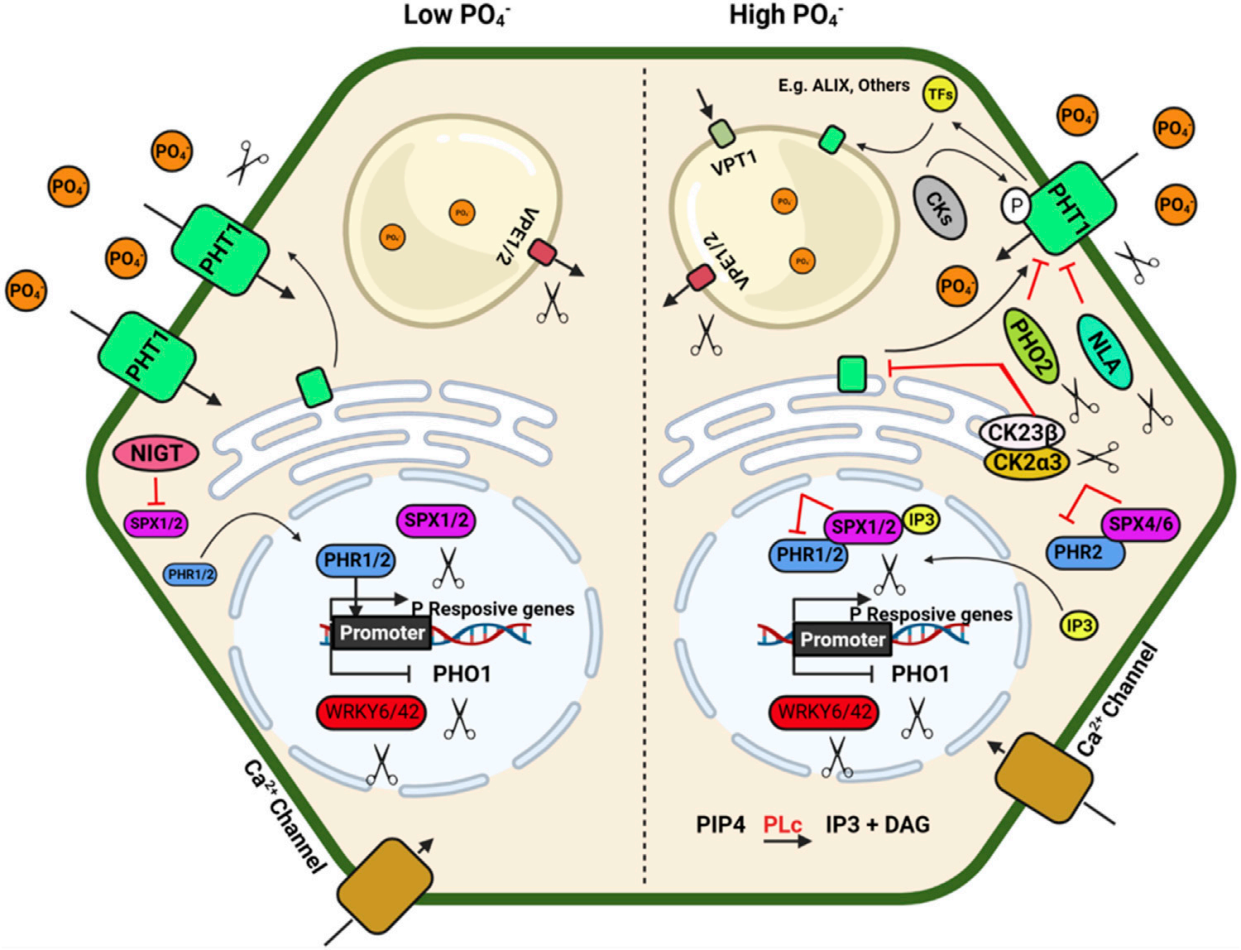
Schematic model depicting plant response to phosphorus availability and signaling. Illustration was made with Biorender (https://biorender.com/). The negative regulators for targeted genome editing are indicated with a scissor sign.

Phosphorus (P) serves is a constituent of ATP, pyrophosphate (PPi), as well as an essential structural component of nucleic acid and phospholipids (Chiou and Lin, 2011; Kumar et al., 2018). P deficiency severely limits crop yield; however, only 10–20% of total applied P fertilizer is used by crops, and the remaining P is unavailable to plants (Oelkers and Valsami-Jones, 2008). The P acquisition efficiency (PAE) and physiological P use efficiency (PUE) depend on the uptake, transport and metabolism driven Pi recycling and overall P homeostasis within the plant (Stigter and Plaxton, 2015; Zeeshan et al., 2020; Prathap et al., 2022). Some of the potential targets and strategies to improve PAE and PUE through genome editing tools are discussed in Figure 2.

The inorganic P (Pi) is immobile in soil and is generally present in the top layer of soil; its acquisition by plants mainly depend on a dense root system with well-developed lateral roots and root hairs (Lynch, 2007; Nestler et al., 2016). A detailed review of root system architecture under P availability and multiple regulatory checkpoints at the molecular level has been described by Niu et al. (2013). Under P deficiency, proteoid or cluster roots (specialized bottle brush-like dense lateral roots) are formed in white lupin, efficiently mobilising Pi from Fe–Pi and Ca–Pi sources. Additionally, some plants have developed a mechanism to enhance Pi availability by secreting organic acids that helps to release Pi from different Pi-containing complexes in soil, which is otherwise unavailable to the plants (Reise and Waller, 2009). Organic acids such as citrate, malate, malonate, and oxalate form stable complexes with metal ions like Al, Fe, and Ca compared to Pi and make free Pi available to the plants (Álvarez-Fernández et al., 2014). Al-activated Malate Transporters (ALMTs) family members play a crucial role in P uptake in soybean and help in K and Fe homeostasis in lupin by regulating malate exudation in acid soil (Liang et al., 2013; Zhou et al., 2020).

P acquisition and homeostasis involve five members of phosphate (PHT) transporters viz., PHT1, PHT2, PHT3, PHT4 and PHT5 in plants (Wang and Wu, 2017) which poses a tremendous opportunity to develop genetically edited crops with high P acquisition efficiency. The plasma membrane-localized PHT1 regulates Pi uptake in roots, while the other PHTs regulate Pi translocation in different cellular organelles. Apart from the PHT family, the PHO1 (an SPX-EXS subfamily) transporter regulates Pi transport from roots to shoot, and VPT1 and OsSPX-MFS1-3 (SPX-MFS subfamily) regulate vascular vacuolar Pi transport (Wang et al., 2018a). The CRISPR knockouts of *SlPH O 1.1* in tomato (Zhao et al., 2019) resulted in a Pi starvation response demonstrated as decreased shoot fresh weight, increased root biomass, and a higher root-to-shoot ratio. These mutants also showed a higher anthocyanin accumulation in the shoot and higher root to shoot soluble Pi content. These results confirmed the importance of *SlPHO1;* 1 gene function in Pi transport in the tomato at the seedling stage. Generation of CRISPR-Cas9 genome-edited plants for the six *PHO1* genes in the tomato genome (*SlPH O 1;1*-*SlPH O 1;6*) will help identify the isoforms’ critical role in P uptake and translocation. The R-type MYB transcription factors PHR1(Phosphate Starvation Response) and PHR2 in Arabidopsis and rice regulate phosphate homeostasis and root hair development. PHR binds to the P1BS (PHOSPHATE STARVATION RESPONSE1 binding site) and helps the plant adapt to Pi deficiency in Arabidopsis. Nuclear localized SPX1/2 and cytoplasmic SPX4 are functional inhibitors of AtPHR1/OsPHR2, a positive regulator of Pi signalling and uptake. N availability regulates Pi uptake and starvation signalling through the NIGT1–SPX–PHR cascade (Ueda et al., 2020). NIGT1 and NIGT1.2 inhibit the expression of *SPX* and thus indirectly activate the *PHR* expression (Poza-Carrión and Paz-Ares, 2019; Ueda et al., 2020; Wang et al., 2020). The *nigt1.1 nigt1.2* double mutant developed by CRISPR-Cas9 displayed reduced P uptake and improved N uptake at low Pi conditions. Other potential negative regulators of Pi signalling are *IPS1/2*, *PHO2*, *AtWRKY6,* ATWRKY43, miR827, etc., affecting multiple downstream genes. Consequently, up-regulation of the different positive regulators of Pi signalling such as *AtPHR1*, *At PHR2*, *PHT1*, *PHO1*, *PAP, SQD2*, *OsSPX*, *MFSs*, *VPT*, *Cm-PAP10.1*, *Cm-PAP10.2*, *Cm-RNS1*, *TaALMT1* and *AVP1*/*AVP1D* etc.) will enhance PUE (Hasan et al., 2016; Wang et al., 2018a).

PHRs also regulate the expression of mi*R399,* which reduces the transcript level of *PHO2*, a negative regulator of Pi deficiency signalling (Bari et al., 2006; Wu and Wang, 2008). CRISPR Cas9 mediated rice mutants *phr1*, *phr2* and *phr3* significantly reduced plant growth. At the same time, double mutants (*phr1/2* *and* *phr2/3*) and triple mutant (*phr1/2/3*) had maximum growth retardation under Pi-sufficient (200 μm Pi) and deficient (10 μm Pi) conditions, respectively. Overexpression of *OsPHR3* improved growth under low-Pi soils and showed average growth under sufficient Pi conditions, implicating positive and diverse regulation of downstream genes for Pi signalling and homeostasis (Guo et al., 2015). In addition to the PHR TFs, several other TFs, namely AtMYB2, AtMYB62, OsMYB2P-1, OsMYB4P, and OsMYB1, are also involved in Pi signalling (Devaiah et al., 2009; Yang et al., 2012, 2014; Gu et al., 2017). Mutation of *MYB1* transcription factor using CRISPR Cas9 system showed an increase in Pi uptake and accumulation (Gu et al., 2017). *MYB1* mutation also altered the expression of multiple genes related to Pi starvation signalling and Pi transporters (*PHT1; 2, PHT1; 9, PHT1; 10, IPS, miR399j,* and *PHO2.1*). A recent study shows that CRISPR-Cas9 double mutant for vacuolar Pi efflux transporters *Osvpe1 Osvpe2* has higher vascular Pi content under low Pi stress than the wild type plants (Xu et al., 2019).

### Genome Editing Targets for Improving Potassium use Efficiency

Potassium (K) is an essential macronutrient for diverse physiological activities during plant growth and development. Fluctuation in external K^+^ concentration generates chemical and physical signals to cope with the imbalanced state of the cellular K^+^ level. These signals directly or indirectly regulate the downstream targets, especially the K^+^ channels and transporters. Thus, plants regulate K^+^ homeostasis to adapt to the varying K^+^ concentration. In plants, K^+^ is involved in multiple physiological activities such as osmotic adjustment, maintenance of membrane potential, ionic balance, enzyme activation, stomatal movement and pollen tube growth (Wang and Wu, 2017). The transporter family HAC/KUP/KT are important for K^+^ transport in plants (Véry et al., 2014). AtAKT1, AtHAK5, and AtKUP7 are major K^+^ transporters in the *Arabidopsis* root system. AtHAK5 is a high-affinity transporter, while AtKUP7 is a low-affinity transporter and mediates K^+^ transport into the xylem and shoots (Han et al., 2016; Nieves-Cordones et al., 2016). In rice, three K^+^ transporters OsHAK1/5/21 have been characterized to transport K^+^ into the root (Yang et al., 2014; Chen et al., 2015). Under deficiency condition, high-affinity transporter OsHAK5 play a role in root and root to shoot K^+^ transport which can be a potent target for genome editing mediated up-regulation (Yang et al., 2014). While OsHAK1 is an interesting transporter with low affinity and high-affinity transport activity (Chen et al., 2015) making it an excellent choice for genome editing techniques. OsHAK16 plays a dual role in K^+^ uptake and translocation and maintain shoot K^+^ homeostasis in rice (Wang et al., 2019c). Members of CHX (cation/H^+^ exchanger), AtCHX14 acts as a K^+^/H^+^ exchanger, a plasma membrane K^+^ efflux transporter. Similarly, other CHXs like AtCHX17 (pre-vacuolar compartment), AtCHX16/18/19, OsCHX14 (ER-localized), etc., are critical regulators of K^+^ homeostasis during different developmental stages (Chanroj et al., 2013; Chen et al., 2016a). In rice, the plasma membrane located cation chloride co-transporter (OsCCC1) transports K^+^ and maintains ion homeostasis and thus play a significant role in cell elongation (Chen et al., 2016b). An *Arabidopsis* the triple mutant of *kea1/2/3* exhibited significantly stunted growth. This is because of their regulatory role in chloroplast development and regulation of pmf (proton motive force) across the thylakoid membrane as a K^+^ transporter (Armbruster et al., 2014; Kunz et al., 2014; Aranda-Sicilia et al., 2016).

Under K^+^ deficiency, CBL1/9-CIPK23-AKT1 and CBL4-CIPK6- AKT2 cascades play important role in maintaining K^+^ homeostasis in plants (Xu et al., 2006; Behera et al., 2017; Cuin et al., 2018; Saito and Uozumi, 2019). The deficiency of K^+^ induces ethylene signalling to generate ROS and regulate the *AtHAK5* transcription level (Schachtman, 2015). Similarly, low K stress decreases cytokinin levels, stimulating ROS production and increasing *AtHAK5* expression (Nam et al., 2012). ROS directly activates Ca^2+^ channels to enhance cytosolic Ca^2+^ levels, activating different K^+^ channels and related transcription factors. Rare Cold Inducible gene 3 (*RCI3*), a type III peroxidase, is also involved in ROS production under K^+^ deficiency (Kim et al., 2010; Demidchik, 2018; Buet et al., 2019). At flowering, jasmonic acid regulates the *OsCHX14* K^+^ transporter gene expression in rice and maintains K^+^ homeostasis (Chen et al., 2016a). Under low K^+^ stress, *OsmIR399* is upregulated and represses the expression of *LTN1/OsPHO2* and indirectly activates the *OsHAK25* transporter (Chen et al., 2015).

**FIGURE 3:**
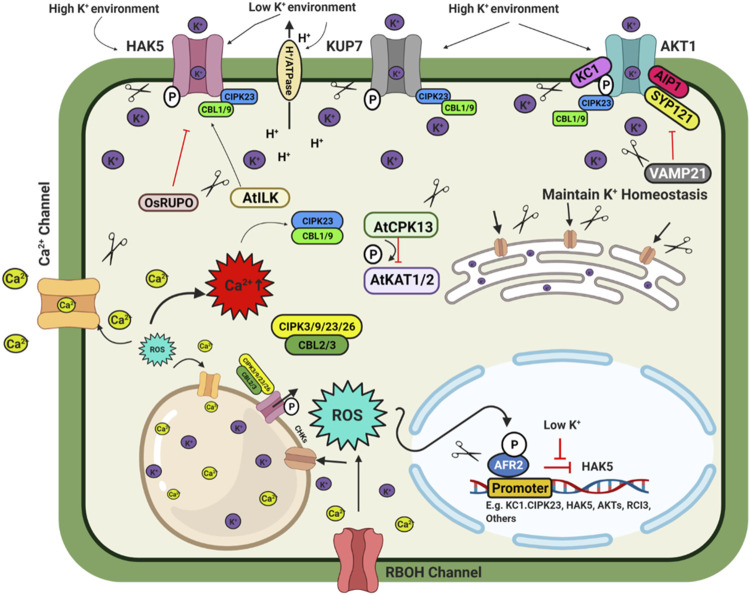
Schematic model depicting plant response to potassium availability and signaling. Illustration was made with Biorender (https://biorender.com/). The negative regulators for targeted genome editing are indicated with a scissor sign.

Transcription factors are essential regulators of the K^+^ homeostasis in plants. Under low K^+^ stress, *AtHAK5* expression increases in Arabidopsis (Gierth et al., 2005). The transcription factor *AtARF2* is a negative regulator of *AtHAK5*. Under sufficient K^+^ conditions, *AtARF2* –TF binds to the promoter of the *AtHAK5* gene and suppresses the expression of *AtHAK5*. K^+^ deficiency leads to phosphorylation of *AtARF2*, and the phosphorylated *AtARF2* dislodges from the *AtHAK5* promoter region and thus permits the expression of the AtHAK5 gene (Zhao et al., 2016). CBL1-CIPK23-AKT1 cascade phosphorylates AKT1 transporter to activate it in *Arabidopsis*, rice, and Venus flytrap (Li et al., 2014; Chen et al., 2015; Ragel et al., 2015). In Arabidopsis, AtCPK13 reduces stomatal opening by phosphorylating AtKAT1/2, inhibiting the inward K^+^ currents and regulating ammonium transporters AtAMT1; 1/1; 2 under high ammonium concentration (Ronzier et al., 2014; Straub et al., 2017). Raf-like MAPKK kinase (*AtILK1*) and rice receptor-like kinase *OsRUPO* also regulate K^+^ homeostasis by activating AtHAK5 accumulation on the plasma membrane and inhibiting K^+^ proliferation by OsHAKs transporters in Arabidopsis and rice, respectively (Brauer et al., 2016; Liu et al., 2016). Rice homozygous *OsRUPO* knockout lines developed by CRISPR-Cas maintained a higher level of K^+^ in pollen than the wild-type plants due to the active OsHAK1. Under normal conditions, RUPO phosphorylates the HAK1 transporter and inhibits the transporter activity (Liu et al., 2016). Besides this TF, some regulatory channel proteins interact with the transporters to modulate their activity. In Arabidopsis channel regulatory subunit AtKC1 and AtCIPK23 synergistically balance K^+^ uptake or leakage and modulate AKT1 mediated low K^+^ responses (Wang et al., 2016). A regulatory SNARE (soluble N-ethyl maleimide sensitive factor attachment protein receptor) protein VAMP721 targets vesicles to the plasma membrane to suppress the AtAKT1-AtKC1 heteromeric channel protein (Li et al., 2017). Nitrate-dependent shoot K^+^ homeostasis is regulated by AtNRT1.5 and OsNPF2.4 in Arabidopsis and rice, respectively (Drechsler et al., 2015; Xia et al., 2015; Meng et al., 2016). Chloroplast localized OsPRX2, a thiol-based peroxidase involved in H_2_O_2_ homeostasis, is a crucial stomatal closure and K^+^ accumulation regulator. Compared to the CRISPR-Cas9 mutated *OsPRX2* lines, *OsPRX2* overexpression lines were more tolerant to K^+^ deficiency tolerance. This result shows that *OsPRX2* is a potential target for genome-editing for K deficiency tolerance (Mao et al., 2018). In rice, OsHAK3 is a K^+^ transporter to maintain K homeostasis. CRISPR Cas9 mediated mutation of *OsHAK3* resulted in lower K^+^ uptake, making them sensitive to low K stress and salinity stress (Zhang L. et al., 2020). By targeted mutation of K^+^ transporter OsHAK1, low Cs^+^ rice was developed (Nieves-Cordones et al., 2017). Above mentioned regulatory checkpoints from K^+^ uptake to transport involving transporters, transcription factors, post-transcriptional and post-translational regulation, etc., are potential targets for improving crop yield under multiple stress conditions and K^+^ limited conditions through genome editing (Figure 3).

### Genome Editing Targets for Improving Iron Use Efficiency

**FIGURE 4:**
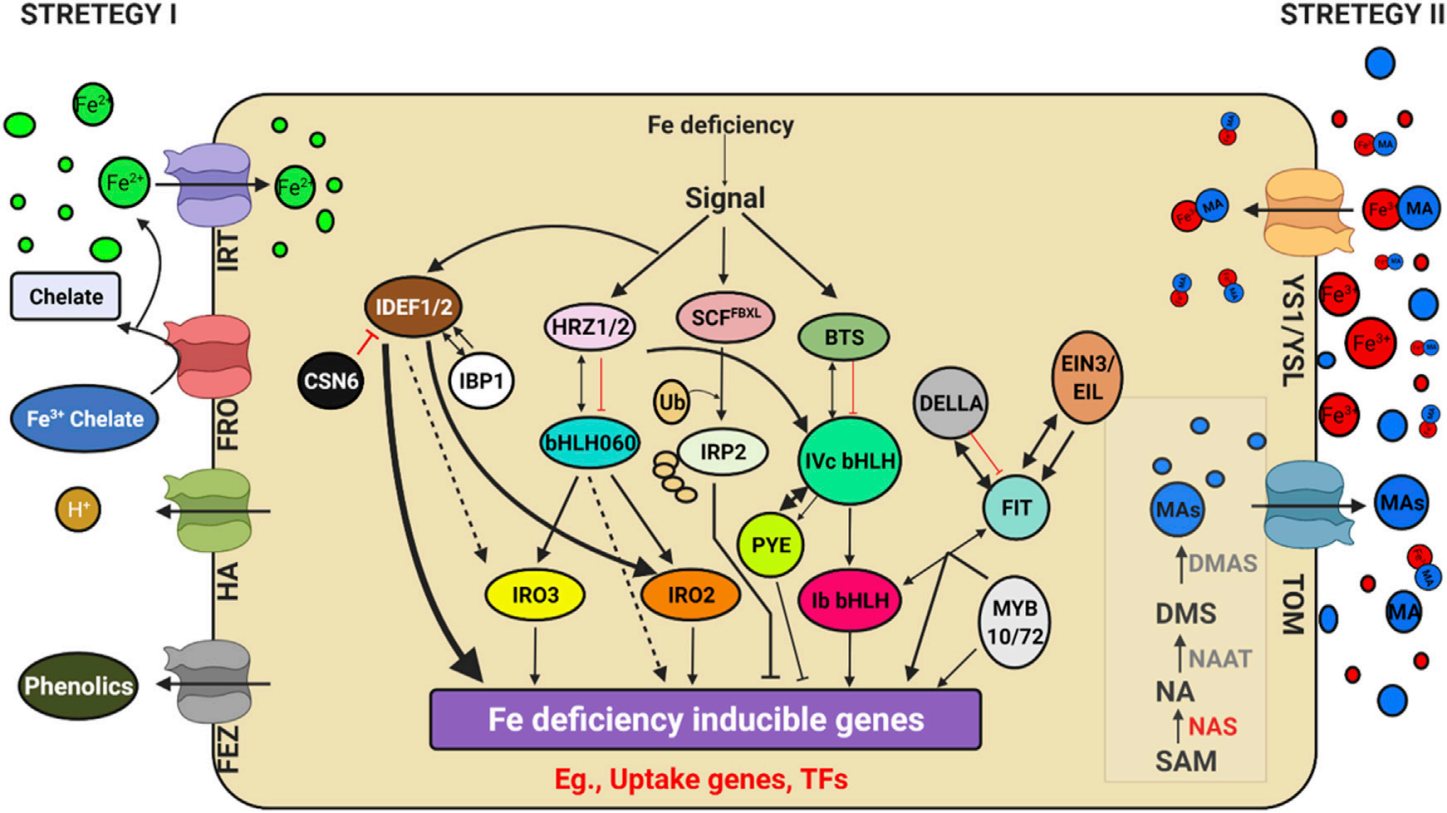
Schematic model depicting Genes regulating iron (Fe) uptake and deficiency response. Illustration was made with Biorender (https://biorender.com/). The possible negative regulators and genome editing targets are indicated with a scissor sign.

Iron is an essential element found in cofactors associated with electron transfer, hydroxylation, and dehydration reactions. Photosystem I and II, ferredoxins, and a variety of metabolic enzymes are particularly rich in heme and iron-sulphur (FeS) proteins (Tissot et al., 2014).) The oxidised condition of Fe in earth crust makes it inaccessible to plants. Iron deficiency led anaemia is a serious human health problem affecting around 30% of the global population, according to the World Health Organization (WHO) (http://www.who.int/nutrition/topics/ida/en/). The Fe deficiency faced by crop plants will thus impact global food and nutritional security. Researchers have discovered a slew of critical factors that regulate iron uptake and metabolism (Figure 4). Traditionally, Fe uptake in plants has been divided into two techniques: strategy I and strategy II, commonly known as reducing and chelating strategies, respectively (Römheld and Marschner, 1986). The fundamental difference is the oxidation state of Fe ion up by the plant: ferrous Fe^2+^ in strategy I and ferric Fe^3+^ in strategy II. In the rhizosphere, Fe is mostly found as Fe^3+^ oxyhydrates with low solubility. Tomato and *Arabidopsis* have been used as models for Strategy I, in which Fe^3+^ is reduced at the plasma membrane by Ferric Reduction Oxidase 2 (FRO2) before being transported across the membrane by Iron-Regulated Transporter 1 (IRT1) (Robinson et al., 1999). AHA2, a plasma membrane proton pump, also aids in acidifying the rhizosphere and increasing Fe^3+^ solubility (Santi and Schmidt, 2009). Grass family (Poaceae) crops were used as a model for strategy II plants, which secrete phytosiderophores (PS), which are tiny organic molecules produced by plants that have a higher affinity for iron molecules (Kanazawa et al., 1994). Deoxymugineic acid is the most abundant phytosiderophore in rice and barley, and it is secreted by TOM1 (Transporter of Mugineic Acid Family Phytosiderophores) (Nozoye et al., 2011). Organic metabolites such as organic acids, flavonoids, phenolics, and flavins were discovered in Strategy I plants. Phenolics were initially thought to help with the solubilization and availability of apoplastic iron until coumarin-related phenolics associated with soil Fe uptake (Lešková et al., 2017). Fraxetin, a major coumarin related phenolic compound, is produced by Feruloyl CoA orthohydroxylase 1 (Fourcroy et al., 2014). Instead of coumarins, alfalfa and sugar beet secrete flavins that aid in the reduction of Fe^2+^ ions (Rodríguez-Celma et al., 2013; Fourcroy et al., 2014). Furthermore, the exudation of putrescine, a polyamine, improved iron mobilisation within the plant cell wall (Zhu et al., 2015). The first report of an oligopeptide transporter (OPT) family protein capable of transporting Fe^3+^-siderophore complex was identified in maize from a mutation in the Yellow Stripe1-Like (YSL) transporter (Curie et al., 2001). Even though non-grasses do not synthesise or secrete PS, many YSL genes have been discovered and are conserved among all land plants. In monocots and dicots, YSLs played a key role in the long-distance transport of metals bound to nicotiamine (NA), such as Fe, Cu, and Zn (Waters et al., 2006), except for the Fe-PS transporters *ZmYS1* (Curie et al., 2009; Lee et al., 2009) and its rice ortholog, OsYSL15 (Inoue et al., 2009; Kobayashi et al., 2014). Eighteen *YSL* genes (*OsYSLs*) have been discovered in rice (Aoyama et al., 2009), and the *OsYSL15* gene has been identified as a Fe-PS transporter involved in Fe acquisition (Inoue et al., 2009; Kobayashi et al., 2014). Under Fe deficiency, the *OsYSL15* gene was highly expressed in root tissues, particularly the epidermis, as demonstrated by OsYSL15 promoter-GUS transgenic experiments (Inoue et al., 2009). YSL1-8 are the eight members of the YSL family identified in Arabidopsis (Jean et al., 2005; Waters et al., 2006). Metal remobilization from senescent leaves is a major role of *YSL* genes, which also serve as transporters in seed development, reproductive organ growth, and long-distance transport of metal complexes with NA (DiDonato et al., 2004). Iron and Cu regulate *AtYSL2* expression, and *YSL2* is abundantly expressed in the root endodermis, pericycle, and xylem cells (Lei et al., 2014). AtYSL2 is thought to play a function in transporting metals to and from the vasculature based on its location. Under control conditions, *ysl2-1* mutants display no apparent characteristics (Schaaf et al., 2005). The *ysl2-1* mutants showed no visual manifestation even when Fe was depleted, showing that additional YSLs must have redundancy activities in transporting metal-NA complexes. Overall, the mutant analysis revealed that FRO2 and IRT1 are required for the uptake of Fe mobilised by coumarins, and that IRT1 is the primary pathway for Fe uptake in Arabidopsis (Shin et al., 2013; Fourcroy et al., 2014; Boonyaves et al., 2016). Furthermore, rice and barley contain a functional homologue of IRT1, which is responsible for facilitating Fe^2+^ absorption in low-oxygen environments (Ishimaru et al., 2006; Boonyaves et al., 2016). Thus, the distinction between strategy I and II is increasingly blurred. In Nature, two opposing Fe^2+^ and Fe^3+^ uptake methods have been observed in the same organism. Fe^2+^ is transferred by the Divalent Metal Transporter DMT1 and Fe^3+^ is captured by transferrin by the same cells as in mammals (Anderson et al., 2013) and Bacteria. Since the discovery of FER, the first critical TF, in tomato (Ling et al., 2002), a plethora of TFs involved in Fe-deficiency response have been identified in *Arabidopsis thaliana* (Hindt and Guerinot, 2012; Lin et al., 2016) and rice (Kobayashi et al., 2014). Because of their functions as potent regulators of Fe deficiency responses and their Fe-binding properties, Iron Deficiency-responsive Element-binding Factor 1 (IDEF1) and Hemerythrin motif-containing Really Interesting New Gene- and Zinc-finger proteins (HRZs)/BRUTUS (BTS) have recently emerged as candidate iron sensors (Kobayashi et al., 2014; Kobayashi and Nishizawa, 2014). IDEF1 is a transcriptional regulator of graminaceous genes involved in Fe absorption and utilisation, which is especially important in the early stages of Fe shortage. Both graminaceous and non-graminaceous plants have HRZs/BTS, which are E3 ubiquitin ligases and negative regulators of Fe deficiency responses. Furthermore, a recent study found that iron man (IMA)/Fe-uptake inducing peptides (FEPs) are positively regulated in plant Fe-deficiency responses (Grillet and Schmidt, 2019; Kobayashi, 2019). Few of these critical regulators are found in both rice and Arabidopsis, and are thus thought to be conserved across plant species, despite differences in downstream genes involved in Fe uptake. The major participants in the transcriptional control of these conserved pathways are basic helix–loop–helix (bHLH) TFs, such as rice OsbHLH060 (OsPRI1) and Arabidopsis AtbHLH34, 104, 105 (ILR3) and 115(Rodríguez-Celma et al., 2013). POPEYE (PYE), is a bHLH TF involved in pericycle-specific iron deficiency response and regulates growth and development in iron-deficient environments (Long et al., 2010). PYE interacts with PYE homologs, such as IAA-Leu Resistant3 (ILR3), a metal ion homeostasis-related bHLH transcription factor. In addition, ILR3 interacts with BRUTUS (BTS), a putative E3 ligase protein with metal ion binding and DNA binding domains, which inhibits the response to Fe. The Fe sensing and signaling molecules upstream of Fe-deficiency responses in plant cells are unclear (Kobayashi, 2019).

### Genome Editing Targets and Case Studies on Improving Salinity and Ion Toxicity Tolerance

Heavy metal toxicity and salinity are major stresses of edaphic origin, detrimental to crop growth and toxic to the animals and humans that feed on them (Munns et al., 2020). Genome editing is a promising tool to enhance the tolerance of crops to salinity and ion toxicity from heavy metal contamination. The research in this regard is evolving faster and has come up with several successful instances.

### Salinity Tolerance

The negative regulators in abiotic stress response pathways are poorly characterised, as a result, there is a scarcity of CRISPR/Cas-mediated studies on abiotic stress tolerance. RECEPTOR FOR ACTIVATED C KINASE 1 (RACK1), a WD40-repeat family protein, is a potent negative regulator of ABA-mediated stress signalling (Guo et al., 2009; Zhang et al., 2013). The constitutive RNAi-mediated knockdown of a RACK1 homolog boosted drought and salt tolerance in soybean plants significantly compared to WT plants (Zhang et al., 2013). The rice RACK1 RNAi lines performed better under drought and salinity stresses, implying that RACK1 is a good target for genome editing. In Arabidopsis, the FARNESYL TRANSFERASE A (FTA) and ENHANCED RESPONSE TO ABA1 (ERA1), which encodes subunits of the enzymatic protein farnesyl transferase that negatively regulate ABA signalling. The knockdown/knockout of both these genes causes ABA hypersensitivity, stomatal closure, and decreased transpiration rates. Mutants of *era1* showed drought resistance and seed dormancy in crops like wheat and rapeseed. These mutants also have enhanced pathogen susceptibility and improved heat tolerance (Wu et al., 2017). Drought-induced down-regulation of the *BnFTA* and *BnERA1* genes in *Brassica napus* has been shown to protect yield under water deficit conditions during blooming while negatively affecting growth under well-irrigated conditions. The ABA hypersensitive Arabidopsis mutants, such as *abh1*, *abo1*, and *cyp85a2*, show improved drought sensitivity; however, less information is available about potential pleiotropic effects or functions in other plant species. Recently it was reported that protein farnesylation reduces the stability of BES1 and negatively regulates the brassinosteroid signalling in *Arabidopsis thaliana* (Zengxiu Feng, 2021). In rice, CRISPR editing of genes associated with carbohydrate metabolism, hormonal homeostasis, and stress signalling gave successful results as compiled in (Ganie et al., 2021).

### Ion Toxicity Tolerance

Excessive accumulation of heavy metals is toxic to crop plants. Metal toxicity severely affects plant growth, development, and yield. It causes oxidative stress and disturbs cellular ionic homeostasis leading to cellular damage. CRISPR-Cas9 system may hold potential for improvement in reducing metal toxicity in plants. The plant genes regulating heavy metal uptake and translocation are potential candidate genes for CRISPR/Cas9 genome editing.

Aluminium toxicity (Al^3+^) is one of the most prevalent toxicity problems in acidic soils. Aluminium inhibits root growth in plants, and the *OsAUX3* (*AUXIN3*) gene plays an important role in the Al sensitivity of rice. *OsAUX3* is an auxin influx transporter that promotes hormone transport acropetally (towards the tips). The *osaux3* mutants generated by CRISPR/Cas9 technology were insensitive to auxin and Al. The defective root growth was less pronounced in the mutants than in the wild type under Al toxicity. The Al accumulation in tissues was also lesser in mutants, as shown by ICP-OES and Morin fluorescence methods (Wang et al., 2019b). Genome editing of positive regulators gave detailed insights into tolerance mechanisms. *ART2* (*Al RESISTANCE TRANSCRIPTION FACTOR 2*) mutant lines in rice were observed to have no pleiotropic effects under normal conditions, but under Al toxicity, the inhibition of elongation was increased (Che et al., 2018). ALMT family of Malate efflux transporters involved in chelating free aluminium in soil solution. The generation of Sl-ALMT9 (*Al-ACTIVATED MALATE TRANSPORTER9*) knockout mutants of tomato resulted in lower root growth and higher Al content in roots, which was evident from hematoxylin staining in root apices (Ye et al., 2017).

The transcription factor OsARM1 (ARSENITE-RESPONSIVE MYB1) is involved in the Arsenic (As)-dependent upregulation of As transport genes in rice. The knockout of OsARM1 resulted in reduced As uptake and tolerance of rice to even higher doses of the heavy metal (Wang et al., 2017). The hydrated diameter of As is similar to P, and hence several phosphate transporters are found to transport the heavy metal. The OsPHF1 (PHOSPHATE TRANSPORTER TRAFFIC FACILITATOR 1) is one such protein having dual specificity. *OsPHF1* mutants generated by EMS treatment led to increased tolerance to As and enhanced biomass even under contamination treatments. The lines had a reduced uptake of both As and Pi (Wu et al., 2011). Thus *OsPHF1* is a promising gene for imparting tolerance against As toxicity via genome editing. Other good targets include *OsPht1;8* (PHOSPHATE TRANSPORTER 1; 8), *Lsi1,* and *Lsi2* (*LOW SILICON RICE 1* and *2*) are significant conduits for As uptake and are early targets for genome editing (Chen et al., 2017). As the knockout of *OsPht1:8*. *Lsi1* and *Lsi2* will influence P and Silicon (Si) uptake, selected alleles specific for As uptake needs to be identified. OsNRAMP1 (Takahashi et al., 2011) transporter responsible for As uptake is also an important genome editing target to reduce AS content in rice.

Os *BET1* (*BORON EXCESS TOLERANT 1*) belongs to the NAC (NAM, ATAF, and CUC) family of transcription factors. It was identified from a RIL derived from IR36 and Nekken 1. The mutated form of the gene led to improved B toxicity tolerance (Ochiai et al., 2011). Another promising target is BnaA9.WRKY47 is a positive transcriptional regulator of B uptake genes in tomatoes. The CRISPR/Cas9 mutants were similar to WT but were sensitive to B deficiency. *Bnaa9.wrky49* mutant line can perform better in B excess soils as the efficient uptake pathway is compromised. The knockout may also lead to such a desirable phenotype of the *BnaA3. NIP5;1* (*NOD26-LIKE INTRINSIC PROTEINS*), the boric acid channel gene downstream of *BnaA9. WRKY47* (Feng et al., 2020).

Iron toxicity is a significant problem in several crops under waterlogged (anaerobic) conditions. Also, Fe, an essential nutrient, must not be prevented from entering the plant. Hence targeting the sequestration and cellular tolerance mechanisms need to be prioritised. Several genes can be targeted to overcome Fe toxicity. *OsFRO1 (FERRIC REDUCTASE OXIDASE 1)* is one such gene that reduces ferric (Fe^3+^) to ferrous (Fe^2+^) form. RNAi-mediated knockdown of OsFRO1 imparted enhanced tolerance to Fe toxicity in rice (Li et al., 2019). Other targetable signalling modules include OsHRZ1 (*HEMERYTHRIN MOTIF-CONTAINING INTERESTING NEW GENE AND ZINC-FINGER PROTEIN 1*), *OsPRI1 (POSITIVE REGULATOR OF IRON HOMEOSTASIS 1), OsIRO2/3 (IRON-RELATED BHLH TRANSCRIPTION FACTOR 2 and 3),* and *NAS1/2 (NICOTIANAMINE AMINOTRANSFERASE 1 and 2)* to develop iron toxicity tolerant rice (Zhang et al., 2017).

Cadmium is a major toxic heavy metal that can enter the food chain, especially through staple foods like rice. OsCCX2/OsCTD1 (CATION/Ca EXCHANGER 2 or CADMIUM TOLERANCE 1) is an efflux channel protein expressed in stem nodes and flowers of rice which transports Cd to aerial parts. The knockout lines exhibited a lower Cd content as well as translocation ratio. The noticeable advantage was that Cd accumulation in the grains was significantly reduced in the mutant lines without compromising yield (Hao et al., 2018). Another Cd transport protein being targeted is OsNRAMP5, whose knockout mutants reduced the metal content in shoots and grains. The inhibition in entry occurred at the roots’ level, and the phenotypic differences were absent between WT and *osnramp5* lines under normal conditions (Tang et al., 2017). In another study by Songmei et al. (2019), mutant lines were generated for OsNRAMP5 (nramp5 × 7 and nramp5 × 9) using CRISPR/Cas9-mediated mutagenesis. Osnramp5 mutants showed low grain Cd accumulation (<0.06 mg/kg). However, only nramp5×7 showed normal growth and yield. Similar results were obtained from knockout lines of the *OsLCT1* (*LOW-AFFINITY CATION TRANSPORTER 1*) gene, expressed in the uppermost node and vascular bundles in rice. In *oslct1* lines, most of the agronomic and yield traits were similar to WT, with 40% lower Cd accumulation (Songmei et al., 2019).

In Arabidopsis, the targeting of *MYB49* led to reduced Cd accumulation. Also, it was found that the abscisic acid-dependent transcription factor ABI5 (ABSCISSIC ACID INSENSITIVE 5) prevents MYB49 activity by direct interaction. It also explains why ABA reduces the uptake of Cd in plants (Zhang et al., 2019). Recently Jia et al. (2022), reported that the knockout of NtNRAMP3 increases Cd tolerance by reducing cytosolic Cd accumulation in tobacco. Specific promising targets to improve Cd tolerance by genome editing include miR390 from rice, a negative regulator of Cd tolerance. In support of this, it was found that the overexpressed lines of miR390 were more sensitive to Cd treatment (Ding et al., 2016). Another set of promising targets is *IRT1* (*IRON-REGULATED TRANSPORTER 1*), *HIPP22, and HIPP44 (HEAVY METAL-ASSOCIATED ISOPRENYLATED PLANT PROTEINs)* from Arabidopsis (Zhang and Reynolds, 2019). From *Triticum turgidum,* the *TtNRAMP6* was also suggested to be a promising target to raise Cd-free wheat (Wang et al., 2019a). In rice root cells, OsNRAMP5 is a key gene involved in controlling the uptake of Cd, Mn, and other metal ions. Knocking out the metal transporter gene OsNRAMP5 by CRISPR/Cas9 reduced Cd accumulation in rice without significantly affecting yield (Tang et al., 2017). Thus, CRISPR-aided genome engineering holds the potential to develop plants with a high level of nutrients along with the reduced amount of toxic metal and anti-nutritional factors for better health.

### Genome Editing Targets and Case Studies on Improving Grain Nutrient Availability and Biofortification

The deficiency of micronutrients or hidden hunger is more pronounced in developing countries. Xie et al. (2015) developed a strategy to produce numerous gRNAs from a single polycistronic gene using an endogenous tRNA-processing system, which precisely cleaves both ends of the tRNA precursor. This system could boost the targeting and multiplex editing capability of the CRISPR/Cas9 system. Using this strategy, three transcription factor genes were targeted (*MADS, MYBR*, and *AP2*) for simplex editing and three other genes (*RPL, PPR*, and *IncRNA*) for multiplex editing. They achieved stable transgenic rice plants with high efficiency (up to 100%). Because tRNA and its processing system are virtually conserved in all living organisms, this method could be broadly used to boost the targeting capability and editing efficiency of CRISPR/Cas9 toolkits. CRISPR-edited plants are free from any foreign DNA; thus, they may have better acceptability compared to traditional GM crops. CRISPR edited mushrooms (Kim and Kim, 2016), false flax with increased oil content, and a drought-tolerant soybean (Waltz, 2018) were able to clear government regulation to reach the market, indicating the potential of CRISPR-edited crops in revolutionizing crop improvement. Developing crop plants with improved nutrient content, reduced toxic metal content, and reduced anti-nutritional factors will benefit better human health. Development of plants with enhanced nutrient content will have a double advantage as improvement in the nutrient content of plants will increase the yield potential, and along with this, it will also increase the flow of nutrients in the food chain as plants are the most important primary producer of our food chain. Genome editing can be exploited to improve the nutritional content of crop plants.

The anti-nutritional factors like phytic acid, protease inhibitors, glucosinolates, lectins, tannins, saponins, amylase inhibitors, reduces bioavailability of nutrients. CRISPR/Cas9 genome editing can be utilized to reduce anti-nutritional factors in plants. In cereal grains, phytate serve as a P store, at the same time, it is considered an anti-nutrient because of its low digestibility. Phytic acid limits the utilization of phosphate as it is present mainly in the organic form of inositol hexakisphosphate (IP6). Hence, low IP6 content can improve crops and grains’ phosphate and mineral bioavailability. Inositol trisphosphate 5/6 kinases (ITPK) enzyme catalyzes the phosphorylation of inositol phosphate to inositol hexakisphosphate, a major phosphate storage form in cereal grains. In Barley, homozygous mutants for the *HvITPK1* gene were generated by CRISPR/Cas9 to elucidate the role of *HvITPK1* in inositol hexakisphosphate synthesis and stress signalling (Vlčko and Ohnoutkova, 2020). The mature grains’ phosphate content was variable, from 65 to 174% compared to wild type (WT). Among 11 mutants highest increase in phosphate content, of 74%, was detected in the homozygous deletion mutant itpk1-14. The insertion mutants showed improved tolerance to salinity stress with a concomitant decrease in grain P content. In wheat the CRISPR/Cas9 editing of *TaIPK1* effectively reduced the phytate content and thereby increased the grain Fe and Zinc (Zn) content (Ibrahim et al., 2022).

Nearly three billion people suffer from deficiency of micronutrients and vitamins, which affects their growth, development, and immunity, increasing the risk for infectious illness. Biofortification, the increase in the content of bioavailable micronutrients in edible parts of staple food crops such as rice, wheat, maize, etc., is considered an effective strategy to provide balanced diets with enriched levels of vitamins and minerals for better human health. The deficiency of micronutrients is attributed to lower uptake of nutrients by plants and accumulation in edible plant parts, presence of high levels of inhibitors affecting their absorption. CRISPR/Cas9 genome editing can be utilized as a powerful tool in manipulating these pathways to increase the micronutrient and vitamin content in plants. In rice and banana, there have been successful attempts to increase carotenoid range by CRISPR/Cas9. Kaur et al. (2018) successfully demonstrated that genome editing through CRISPR/Cas9 can be applied as an efficient tool for banana genome modification by creating mutation in phytoene desaturase (RAS-PDS) of banana cv. Rasthali. Further, in 2020, they developed the β-carotene-enriched Cavendish banana cultivar (cv.) Grand Naine (AAA genome). In the carotenoid biosynthesis pathway, lycopene is bifurcated by lycopene cyclases, i.e., lycopene beta-cyclase (LCYβ) and lycopene epsilon-cyclase (LCYε) into β-carotene and α-carotene, respectively. A low activity level of lycopene epsilon-cyclase will increase the availability of lycopene for β-carotene. Thus, the Lycopene Epsilon-Cyclase (LCYε) gene was targeted for CRISPR gene editing. Point mutations/premature termination of LCYε protein multiple types of indels in the genomic region of Grand Naine LCYε (GN-LCYε) were obtained. In edited plants the β-carotene content was improved by 6-fold (∼24 μg/g) in comparison to non-edited plants (Dong et al., 2020). These results demonstrate that CRISPR-Cas9 genome editing is a promising strategy for the genetic improvement of rice and other crops. The targeted gene expression strategy can be utilised to insert genes involved in the uptake and translocation of micronutrients to improve their content in plants.

The sgRNA guided genome editing system, using type II CRISPR/Cas9, has been demonstrated by targeting the *Phytoene desaturase* (*ClPDS*) gene to create knockout mutations in *C. lanatus* (Tian et al., 2017). The *ClPDS* gene is essential for chlorophyll biosynthesis and acts as a critical enzyme in the biosynthesis of carotenoids (Tian et al., 2017). The phytates in protein storage bodies chelate with several mineral cations, including Zn^2+,^ Fe^2+^, Ca^2+^, and Mg^2+^ in grains. During seed germination to utilize the stored mineral ions in developing seedling, endogenous grain phytase is activated to degrade phytate, releasing myo-inositol, P, and bound mineral cations. The crop plants with low levels of IP6 content may have higher phosphate and mineral bioavailability. The inositol trisphosphate five and inositol trisphosphate six kinases enzymes (ITPK’s) participate in the sequential phosphorylation of inositol phosphate to inositol hexakisphosphate, an effective phosphate storage form in cereal grains. In Barley, homozygous mutants for the *HvITPK1* gene were generated by CRISPR/Cas9 to elucidate the role of HvITPK1 in inositol hexakisphosphate synthesis and stress signalling (Vlčko and Ohnoutkova, 2020). The mutation in *HvIPTK1* altered phosphate levels from 65 to 174% in the mature grains compared to wild type content. Among 11 mutants highest increase in phosphate content, of 74%, was detected in the homozygous deletion mutant *itpk1-14*. On the contrary, mutant insertion lines revealed a higher tolerance to salinity stress than deletion mutants and reduced grain P content.

Nucleotide substitutions of *OsITPK6* could significantly reduce rice grain phytic acid content. Jiang et al. (2019) targeted the first exon of *OsITPK6* using the CRISPR/Cas9 method. In the four OsITPK6 mutant lines, one (*ositpk6_1*) is with a 6-bp deletion (no change in frame), and the other three with frameshift mutations (*ositpk6_2, _3, and _4*). In frameshift mutant lines, plant growth and reproduction were severely impaired. At the same time, the effect of the in-frame mutation in *ositpk6_1* was relatively limited. The mutant lines *ositpk6_1* and *6_2* had significantly low phytic acid content and higher inorganic P levels than the WT.

Researchers utilised the CRISPR/Cas9 technique to engineer browning genes in mushrooms, where minimising browning in white truffles led to the extension of their life span, thereby offering a business advantage. The deletion mutant of the polyphenol oxidase gene reduced the oxidation of polyphenols when exposed to air, bringing an appetising and attractive appearance to the mushrooms (Waltz, 2018). Similarly, the mutation in the gene that encoded the polyphenol oxidase (PPO) enzyme in apples led to a reduction in browning in cut apples (Nishitani et al., 2016). Other workers reported the technology application by developing acrylamide free potatoes (Halterman et al., 2016) and low phytic acid with higher available P content corn line (Liang et al., 2014). Rice, a principal food crop, has been the primary target for quality enrichment. Amylose is considered a vital nutritional quality parameter of rice grains. Targeted editing of the starch branching enzymes produced mutant lines with lower amylose content in rice grains (Sun et al., 2017). In soybean, targeted modification of the omega-6 desaturase (*GmFAD2*) gene via the CRISPR/Cas9 method resulted in the higher accumulation of oleic acids via a reduction in the linoleic and a-linolenic acids (Al Amin et al., 2019).

### Strategies to Exploit Potent Regulatory Genes for Improving NtUE

Single-nucleotide polymorphisms regulate agronomically important traits, including the determinants of NtUE. Base editing and prime editing techniques are recently developed precise genome editing techniques which can generate/correct a point mutation without inducing a DSB. The base editors are classified as cytosine base editors (CBEs; C: G to T: A) and adenine base editors (ABEs; A: T to G: C). Cytosine base editors consist of dead Cas9 (dCas9)/Cas9 nickase, cytidine deaminases, and a uracil DNA glycosylase inhibitor. In ABEs, cytosine deaminase is replaced by a mutant *Escherichia coli* transfer RNA adenosine deaminase (TadA). The sgRNA guides the CBE system to the target region of DNA and induces R-loop formation. Then, the cytidine deaminase deaminates the cytosine into uracil in the non-target DNA strand, and subsequent DNA repair and replication results in cytosine to thymine base conversion (Jiang et al., 2020). In ABEs, adenine is deaminated into inosine in the non-target strand, and subsequent DNA repair and replication cause adenine to guanine base conversion. The CBE system was first established for plants by creating a point mutation in the nitrate transporter NRT1.1B and Slender Rice 1 (SLR1, a DELLA repressor) gene in rice plants (Lu and Zhu, 2017). Recently a new TadA variant, TadA9 was identified, which has high-efficiency, multiplex adenine base editing and compatible with CRISPR/SpCas9, CRISPR/SpCas9-NG, CRISPR/SpRY and CRISPR/ScCas9 nickase thus enhancing its editing window at diverse PAM sites. The identification of class 2 CRISPR effector proteins, Cpf1 (Clustered regularly interspaced short palindromic repeats from prevotella and francisella 1) lead to the development of an advanced genome editing system, i.e. CRISPR/Cpf1. This new system of base editors (CRISPR/Cpf1) is evolved as more accurate and efficient genome editing as they have the advantages of low molecular weight and reduced off-target activity (Alok et al., 2020). The reduced size of Cpf1 proteins compared to Cas9 makes it possible to design smaller vectors and better transformation efficiency.

Prime editing technique is also known as “search and replace genome” editing technique (Lin et al., 2020). The prime editing guide RNA (pegRNA) containing a desired edits is fused to Cas9 nickase and reverse transcriptase (RT). The pegRNA has a primer binding site and an RT template including edited nucleotides. The Cas9 nickase and RT complex is guided by the pegRNA to the target DNA followed by Cas9 mediated nick formation. The reverse transcriptase then uses the edited pegRNA as a template and starts reverse transcription from the nick site. The newly edited DNA strand replaces the original DNA strand and makes a hetero-duplex of one edited and one unedited DNA strand. In the final stage, the edited DNA strand acts as a template over non-edited DNA strand and the DNA repair mechanism repairs the non-edited DNA strand. Prime editing holds great promise for precision breeding for developing superior crops for traits, such as increasing yield, stress tolerance and resource use efficiency.

CRISPR/dCas9 system also offers the added advantage of exploiting transcriptional activators/repressors for overexpression of genes of interest in a targeted manner. CRISPR activation (CRISPRa) and CRISPR interference (CRISPRi) are variants of CRISPR in which a dCas9 dependent transcription activation or interference is achieved. To improve guide RNA efficiency, specialized peptide epitopes are fused to the dCas9. The recently developed dCas9-SunTag system (Tanenbaum et al., 2014), was employed to engineer targeted gene activation and DNA methylation in Arabidopsis (Papikian et al., 2019).

Cis-engineering is observed to be a more promising way to regulate the gene expression because of lower detrimental pleiotropic effects in comparison to coding sequences (Pandiarajan and Grover, 2018; Wolter and Puchta, 2018). But very limited research has been carried out in plants where target gene expression was regulated by insertion of new cis regulatory element (CRE) or disruption/deletion of existing CREs. One such example of deletion was studied in rice where disease resistance was improved by regulatory fragment deletion spanning 149bp which included a transcription-activator-like effector (TALe)- Binding Element (EBE) in SWEET11’s (SUGARS WILL EVENTUALLY BE EXPORTED TRANSPORTERS 11) promoter (Li et al., 2020). Insertion of transposable elements (TEs) can alter the expression of desirable genes. In tomato, the insertion of a transposable element in tomato homolog of *SEPALLATA4* and 564 bp in *FRUITFULL* homolog improved floral architecture along with higher fruit weight, number and yield (Soyk et al., 2017). Gain of function of alleles can also be obtained by cis-engineering involving CRISPR/Cas to alter CREs of introns and other downstream genes. One notable example is CArG element disruption downstream of SlWUS including two SNPs (Li et al., 2018b).

Promoter insertion and swapping can potentially be used to improve nutrient use efficiency which can be achieved by HDR. Under drought conditions maize yield was improved by moderate overexpression of *AGROS8,* an ethylene response negative regulator using site-specific insertion in 5′ UTR using Cas9 induced DSBs (Shi et al., 2017). Additionally, the epigenome can also be edited using CRISPR/Cas mediated cis-engineering. Although a very limited attempt has been made to describe epigenome editing involving alteration in histone acetylation (Roca Paixão et al., 2019) and DNA methylation (Gallego-Bartolomé et al., 2018; Papikian et al., 2019). Upstream open reading frames (uORFs) could be used to improve nutrient use efficiency. uORFs are protein-coding short elements localised in the 5’ leader region which controls the quantity of downstream primary ORFs (pORFs) which are synthesised (von Arnim et al., 2014). In plants, the uORFs occupy 30–40% of the transcript proportion(von Arnim et al., 2014). Previously rice plant immunity was improved by the insertion of uORF upstream of pORF without yield reduction (Xu et al., 2017). To date, very limited research was conducted to improve nutrient use efficiency using CRISPR/Cas genome editing targeting positive regulators.

Transporters are one of the important positive regulators whose activity can be enhanced using CRISPR/Cas based genome editing. One attempt has been made to improve the activity of OsNRT1.1B using dCas9 (D10A) fused with rat’s cytidine deaminase enzyme APOBEC1 to replace C to T resulting in Thr327Met mutation. Though improvement in N use efficiency was not tested (Lu and Zhu 2017). Some key changes can enhance the activity of transporter viz., Tyr312Asp in PHT1; 1 for improved Pi transport (Fontenot et al., 2015), Phe130Se in HAK5 to increase the affinity to K^+^ (Alemán et al., 2014). Nitrate transport can be increased by enhancing the activity of NRT1s and NRT2s. CRISPR activator led increase in binding of TFs NF-Y, NAC2, MADS57 and NLP4 may enhance the nitrate transport (He et al., 2015; Qu et al., 2015; Huang et al., 2019; Wu et al., 2020). The expression of *NRT1* can be augmented by increasing the CAT box and CArG motifs in the NRT1 promoter (Qu et al., 2015; Huang et al., 2019). Phosphate transport can be improved by amplifying the PHT1 activity which can be achieved by CRISPR activator led enhancement in the binding of PHR1 to P1BS, MYCS to P1BS (Chen et al., 2011). PHO2 negatively regulates the PHT1 activity involving ubiquitination (Liu et al., 2014). SPX1,2 of rice also acts as a negative regulator of PHT1 (Zhou et al., 2015; Gu et al., 2016). Repression of these negative regulators by dCas9 with specific gRNA may improve P transport. W-Box, P1BS, MYCS and other motifs can also be engineered to enhance Pi transport in plants (Ceasar et al., 2022). The expression of the *AMT1* plant gene family may be upregulated by enhancing the binding of IDD10 to DOF18 TFs through a CRISPR activator (Xuan et al., 2013; Wu et al., 2017). Further CRISPR activator mediated binding of RAP2.11, TFII_A, bHLH121, DDF2 and JLO TFs could amplify the expression of *HAK* transporter. GCC-box and AuxREs motifs can be engineered into the promoter of HAK to enhance its expression through CRISPR prime editing (PE) (Hong et al., 2013). bZIP19 and 23 binding to ZDRE can be complemented by CRISPR activator while CRISPR PE could be employed to increase ZDRE motifs elicitation of *ZIP* genes (Ceasar et al., 2022). Similarly other nutrient transcriptional factors and motifs could be engineered to improve the nutrient use efficiency.

## Summary and Conclusion

**FIGURE 5:**
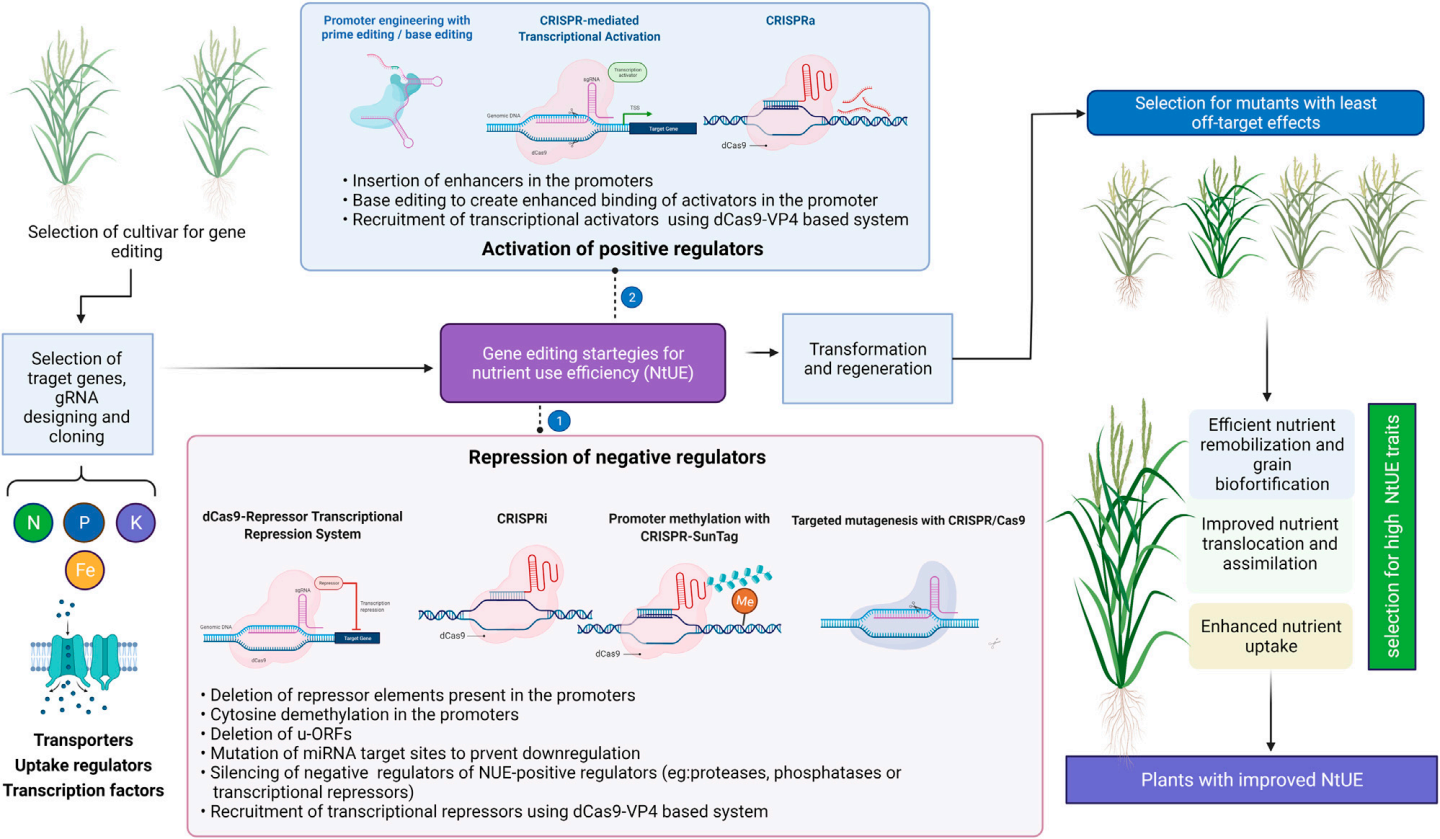
Gene editing strategies for improving nutrient use efficiency (NtUE). The approaches to utilize the positive and negative regulators of the trait of interest are presented. Illustration was made with Biorender (https://biorender.com/).

CRISPR/Cas9 has evolved as cutting-edge technology for better understanding of gene functions, characterising molecular networks, and improving the yield, nutritional content, NtUE, and tolerance to biotic and abiotic stresses. A customized small sgRNA directs Cas9 nuclease at a specific genomic location. Cas9 makes a double-stranded break three to four bp upstream to the PAM site at the target site. Subsequently, the cell’s natural repair mechanism of HDR or NHEJ repairs double-strand breakage (Miglani, 2017). As NHEJ repair is error-prone, it creates random insertions/deletions and indels, resulting in frameshift mutations and targeted gene knockouts (Jinek et al., 2012a; Feng et al., 2013). Compared to earlier genome editing methods, i.e. zinc-finger nucleases and transcription activator-like effector nucleases, the CRISPR/Cas9 offers the accurate and efficient targeted modification in the genome of any organism in a simple way. The specific genome editing targets for improving nutrient response and nutrient stress tolerance are compiled in Table 1. The major genome editing strategies for improving NtUE is presented in Figure 5. Further development in the CRISPR/Cas9 system allows for the editing of multiple genes at one time (Donohoue et al., 2018), and mutations can be targeted to the untranslated region of coding genes (Mao et al., 2018), promoter regions (Seth, 2016), microRNAs (Chang et al., 2016), non-coding RNAs (ncRNAs) (Wang et al., 2018c). CRISPRi and CRISPRa are among the development of technology in which transcription factors are fused with dCas9 to repress or enhance transcription by RNA polymerase and subsequently, upregulate or downregulate the expression of a gene/genes of interest. Researchers can effectively use the knowledge of genes regulating nutrient homeostasis in plants and advanced techniques in genome editing to design plants with desired traits. The development of resource use efficient, high NtUE plants through CRISPR-Cas technology will enhance the pace of genetic improvement for yield.

**TABLE 1:** Compilation of genome editing case studies and targets for improving nutrient response and toxicity tolerance.

Trait	Genome editing targets	Functions	Effects of targeted editing or RNAi suppression	References
Nitrogen use efficiency	*LATERAL ORGAN BOUNDARY DOMAIN (LBD) gene family transcription factors (LBD37, LBD38, and LBD39)*	Repressors of anthocyanin biosynthesis and nitrate-responsive genes including NRT2.1, NRT2.2, NIA1, and NIA2		Rubin et al. (2009)
	*SPL9 At upstream*	Reduces the transcript levels of NRT1.1, NIA2, and NIR.	—	Krouk et al. (2010)
	*Leaf width 5 (LW5)*	LW5 is an allele of D1, encoding the rice G protein α subunit	The loss of LW5 leads to an increase in photosynthesis, N uptake, and chlorophyll content	Zhu et al. (2020)
Phosphorous use efficiency	*MYB1*	Negative regulator of Pi starvation signalling and Pi transporters (*PHT1;2, PHT1;9, PHT1;10, IPS, miR399j* and *PH O 2.1*)	Mutation of *MYB1* transcription factor using CRISPR Cas9 system showed an increase in Pi uptake and accumulation	Gu et al. (2017)
Salinity tolerance	*RECEPTOR FOR ACTIVATED C KINASE 1 (RACK1)*	Negative regulator of ABA responses in plants and	Constitutive RNAi-mediated down-regulation of RACK1 homolog in soybean has been found to increase drought and salt tolerance	Zhang et al. (2013), Chen et al. (2018), Zheng et al. (2019)
	*STRESS-ASSOCIATED PROTEIN 1(SAP1)*	—	Down-regulation of PagSAP1 in poplar enhances salinity tolerance by an increase in stress response genes	Yoon et al. (2018)
	*OsRR22*	Transcription factor involved in cytokinin signal transduction and metabolism	Loss of function and CRISPR/CAS mediated editing of OsRR22 resulted in salt tolerance in rice	Takagi et al. (2015), Zhang et al. (2019)
Metal toxicity	*OsAUX3*	Auxin influx transporter	The *Osaux3* mutants generated by CRISPR/Cas9 were insensitive to Al and accumulated less Al in tissues under Al toxicity	Wang M. et al., 2019
	*OsARM1 (ARSENITE-RESPONSIVE MYB1)*	—	CRISPR/Cas9 based genome editing of *OsARM1* resulted in reduced As uptake and As tolerance of rice	Wang et al. (2017)
	*BET1 (BORON EXCESS TOLERANT 1)*	—	Loss of function mutation improved B toxicity tolerance	Ochiai et al. (2011)
	*OsCCX2/OsCTD1 (CATION/Ca EXCHANGER 2 or CADMIUM TOLERANCE 1)*	Cadmium efflux channel protein expressed in stem nodes and flower of rice transports Cd to aerial parts	The knockout lines showed a lower Cd content	Hao et al. (2018)
	*OsFR O 1 (FERRIC REDUCTASE OXIDASE 1)*	Reduces ferric (Fe^3+^) to ferrous (Fe^2+^) iron	RNAi mediated knockdown of *OsFRO1* imparted enhanced tolerance to Fe toxicity in rice	Shou et al. (2019)
Biofortification	*OsNramp5*	Transport of Cadmium (Cd) to grains	CRISPR/Cas9 mediated knocking out of metal transporter *OsNramp5* it reduced Cd accumulation in rice grains	Tang et al. (2017)
	*OsITPK6* (Inositol trisphosphate kinase 6)	ITPK enzymes catalyse the sequential phosphorylation of inositol phosphate to inositol hexakisphosphate/phytic acid, the primary storage form of phosphate in cereal grains	CRISPR/Cas9 mutant lines *Ositpk6_1* and *Ositpk6_2* had low phytic acid and higher levels of grain P	Jiang et al. (2019)
	*GmFAD2*		Targeted modification of the GmFAD2 by CRISPR/Cas9 increased the accumulation of oleic acids and reduced the linoleic and linolenic acids	Al Amin et al. (2019)
